# Uncertainty Quantification for Mechanical Properties of Polyethylene Based on Fully Atomistic Model

**DOI:** 10.3390/ma12213613

**Published:** 2019-11-04

**Authors:** Nam Vu-Bac, X. Zhuang, T. Rabczuk

**Affiliations:** 1 Cluster of Excellence PhoenixD (Photonics, Optics, and Engineering—Innovation Across Disciplines), 30167 Hannover, Germany; 2 Division of Computational Mechanics, Ton Duc Thang University, Ho Chi Minh City, Vietnam; 3 Faculty of Civil Engineering, Ton Duc Thang University, Ho Chi Minh City, Vietnam

**Keywords:** uncertainty quantification, fully atomistic model, mechanical properties, bayesian updating, Kriging

## Abstract

This study is to assess the effect of temperature and strain rate on the mechanical properties of amorphous polyethylene (PE) based on fully atomistic model. A stochastic constitutive model using data obtained from molecular dynamics (MD) simulations for the material is constructed. Subsequently, a global sensitivity analysis approach is then employed to predict the essential parameters of the mechanical model. The sensitivity indices show that the key parameter affecting Young’s modulus and yield stress is the temperature followed by the strain rate.

## 1. Introduction

Due to exceptionally physical and mechanical properties, polymers, which are considered as new class of lightweight materials, are extensively used in the automotive, aerospace industry etc. Several studies have been made to gain comprehensive understanding complex mechanical behavior of the materials, especially, temperature and strain rate dependent Young’s modulus and yield stress. In order to predict the mechanical properties over a large range of conditions, e.g., temperatures, strain rates, etc., molecular mechanism associated with nanostructure of the polymers must be investigated. Since experiments for nanomaterials are expensive, challenging and sometimes impractical, computational methods can be employed as a replacement method to predict the material behavior. Molecular dynamics (MD) simulations were employed by Vu-Bac et al. [1] to construct the constitutive laws for polymers within the multiscale modeling framework. In this study, a united atom (UA) model was used to simulate the viscoplastic behavior of polyethylene (PE) material. However, to describe influence of specific atoms on the mechanical response of materials accurately, a comprehensively understanding of their atomic details, chemical structure is required. Fully atomistic models thus should be used to gain the above-mentioned structure-property relationship. As pointed out in [2], MD simulations using fully atomistic model allows us to approach closer the realistic yielding mechanism of highly cross-linked epoxy.

Since viscoelastic-plastic behavior is of paramount importance for polymers, the temperature and strain rate dependent mechanical properties such as Young’s modulus, yield stress have been qualitatively examined. However, the temperature and strain rate were considered as deterministic parameters in previous studies [3]. This may result in an overestimation of the mechanical model. Stochastic modeling instead should be used where the model parameters are treated as random field.

This study is to address stochastic constitutive law for the PE material whose uncertain parameters have been taken into account. The material is simulated based on fully atomistic model. Tensile strain are simulated on the PE’s systems over a wide range of temperature below glassy transition state ($T_{g}$) and loading rate. The uncertain temperature and strain rate effect on the Young’s modulus and yield stress then have been examined. The uncertainty quantification serving as a validation function has also been conducted to estimate the influence of those uncertain parameters on the mechanical properties.

The article is organized as follows. In the next section, we briefly depict nanoscale model with the fully atomistic structure, force field and the simulations’ results. Stochastic modeling is depicted in Section 3. A description of surrogate models addressing the mechanical properties dependence on the temperature and strain rate is presented in Section 4. Section 5 presents implementation of global sensitivity analysis (SA). Subsequently, numerical results are discussed. Finally, we close the manuscript with concluding remarks.

## 2. Nanoscale Model

### 2.1. Model System and Molecular Force Field

**Figure 1:**
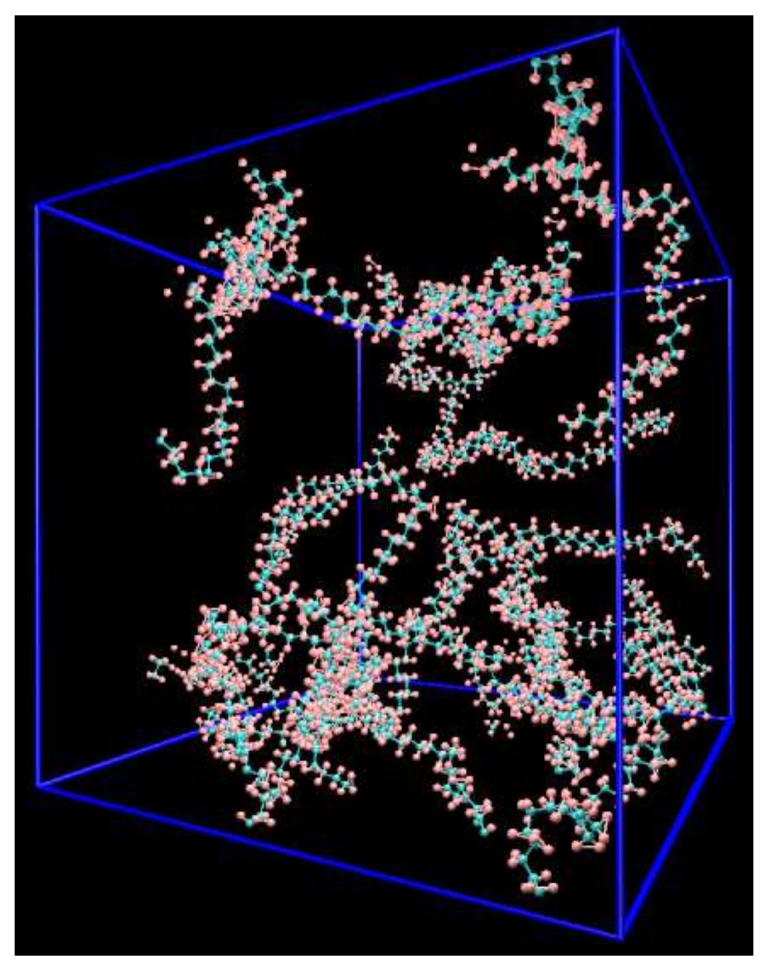
System of PE consisting of 40 PE molecules.

The initial structure of a polymer consists of 40 PE molecules (200 Carbon (C) atoms on the backbone) which were randomly seeded in a 3D periodic cubic box as shown in Figure 1. In this study Monte-Carlo self-avoiding random walks method is used to generate the initial PE system. At first, a simulation box is generated based on a face-centered cubic (FCC) grid. Then, C-atoms on polyethylene backbones are added to the lattice in a step-wise manner. Particularly, an initial atom is located randomly at a site on the lattice and then the polymer chain is grown in each possible direction where sites are available in accordance with a probability. Details of the method can be found in Binder [4]. This structure was then equilibrated by LAMMPS package [5] through four sequential steps: Firstly, the PE systems were equilibrated for $10^{5}$ timesteps (Δt=1) fs at 500 K using a Nose-Hoover thermostat (NVT) dynamics [6,7]. Next, a Nose-Hoover barostat (NPT) that maintains the temperature at 500 K and the pressure at 1 atm was conducted for $5\times 10^{5}$ timesteps (Δt=1 fs). The structure was then cooled down to the desired temperature with a cooling rate of 0.4 K/ps followed by further equilibration to bring the structure to its equilibrium state for $5\times 10^{5}$ timesteps at the same temperature.

The DREIDING force field [8] with harmonic covalent potential functions is used to describe the PE material modeled in this study. Buckingham functions (exponential-6) is employed to explain van der Waals interactions. Also, self-consistent computations using the electronegativity equalization method are used to gain the atomic charges.

**Figure 2:**
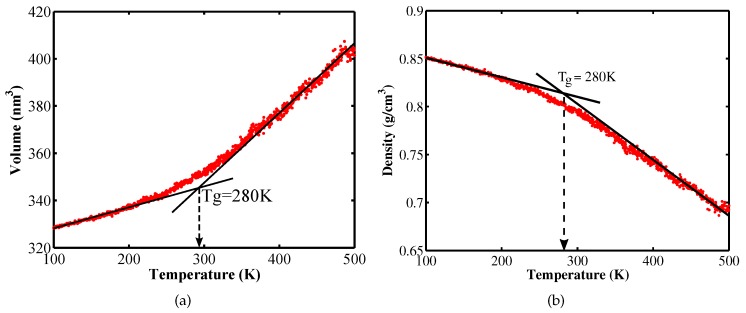
The change in specific volume (**a**) and density (**b**) as a function of temperature.

In order to plot the volume evolution as a function of the temperature during equilibration process from which the glass transition temperature can be determined we perform the follwing steps: At first, the systems was equilibrated at 500 K and then cooled down to 300 K for $5\times 10^{5}$ equilibration steps (with the cooling rate of 0.4) K/ps. Figure 2 shows plots of the volume and density changes during cooling down process as functions of the temperature. The glass transition temperature $T_{g}$ is identified as the intersection of to linear fitted lines of the MD volume-temperature data, see Figure 2. The glass transition temperature $T_{g}=280$ K obtained from simulations is in good agreement with the experimental value $T_{g}=250$ K reported in [9] and $T_{g}=255$ K in [10].

### 2.2. Deformation Simulations

**Figure 3:**
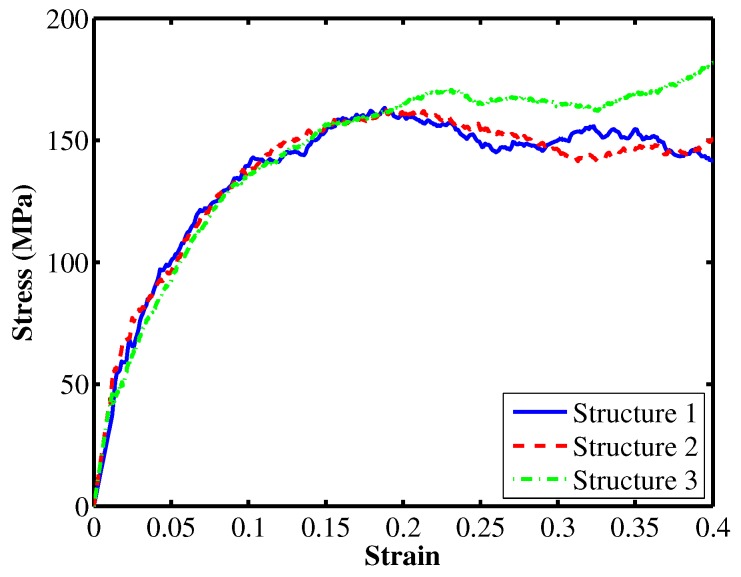
Stress-strain responses corresponding to three different initial PE systems at temperature of 250 K.

**Figure 4:**
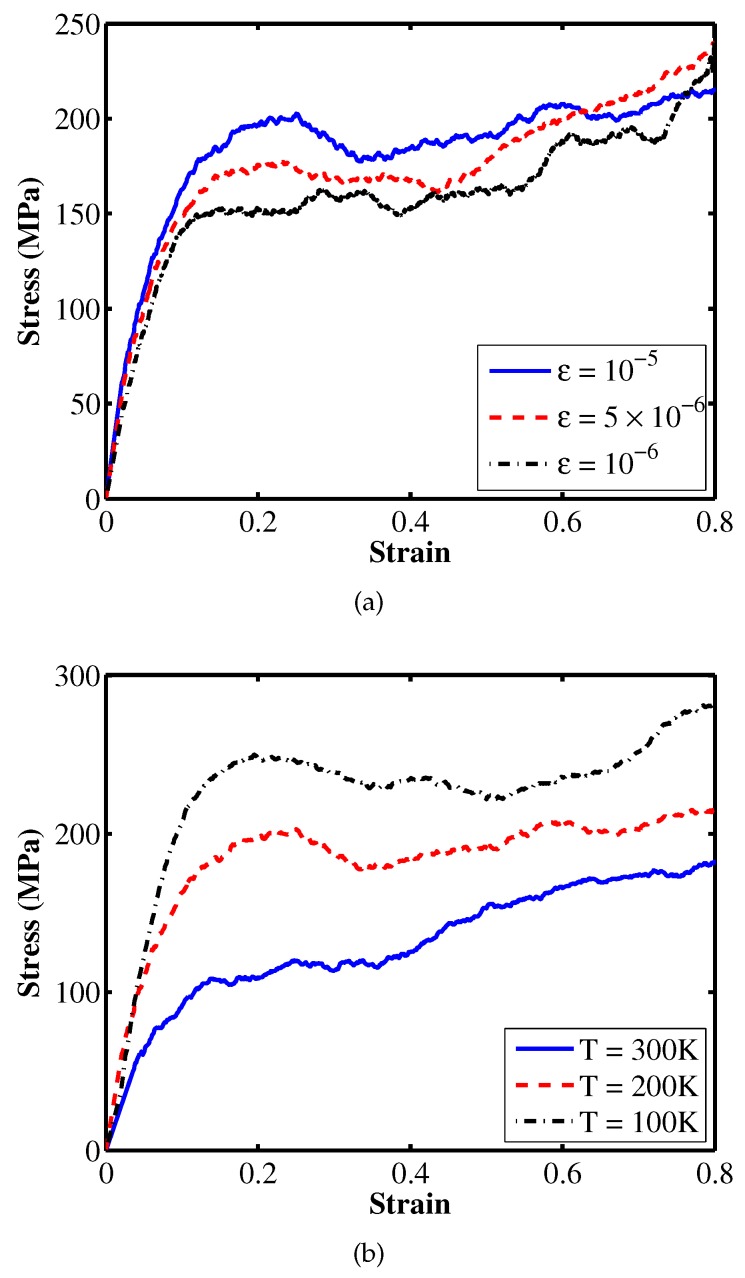
Uniaxially tensile stress-strain responses for the PE deformed (**a**) at temperature of 200 K for different strain rates; (**b**) at a strain rate of $10^{-5}$ 1/fs for different temperatures.

Once the system obtains the equilibrium state, we apply a uniaxially tensile strain to measure the tensile stress-strain response of the material. The system is deformed under the NPT dynamics with different temperatures and strain rates. Figure 4a shows the tensile stress-strain curves at temperature of 200 K for different strain rates while the stress-strain curves at strain rate of $10^{-5}$ 1/fs for different temperatures. Results obtained from MD simulations are shown in Table 1. The tensile Young’s modulus is 1.22 GPa corresponding to the room temperature of 300 K at a strain rate of $1\times 10^{-5}$ 1/fs, i.e., the blue solid curve in Figure 4b. These results are in good agreement with experimental results shown in [11]. The stress-strain curves at 250 K corresponding to three different initial structures, which are generated randomly, are examined. The obtained Young’s modulus and yield stress are nearly independent of the initial configurations as shown in Figure 3. Figure 4 depicts uncertainty as a function of temperature and strain rate within nanoscale model.

The method employed by Theodorou et al. [12] for the quasi-static modeling can be summarized by the following steps: (1) Given an undeformed structure in equilibrium state whose continuation vectors $a_{x0},\;a_{y0}\;\mathrm{and}\;a_{z0}$ are mutually perpendicular and equivalent in magnitude. (2) An uniaxial tension is then applied, e.g., in *x* direction, the continuation vectors hence become $a_{x}=\left(1+\varepsilon \right)a_{x0}$, with ε being the applied strain while $a_{y}$ and $a_{z}$ are kept fixed. (3) Subsequently, the total potential energy is minimized using the BFGS quasi-Newton algorithm with the continuation vectors are kept constant to their values in deformed configuration. We iterate step 2 followed by step 3 consecutively until the desired degree of deformation can be gained.

**Figure 5:**
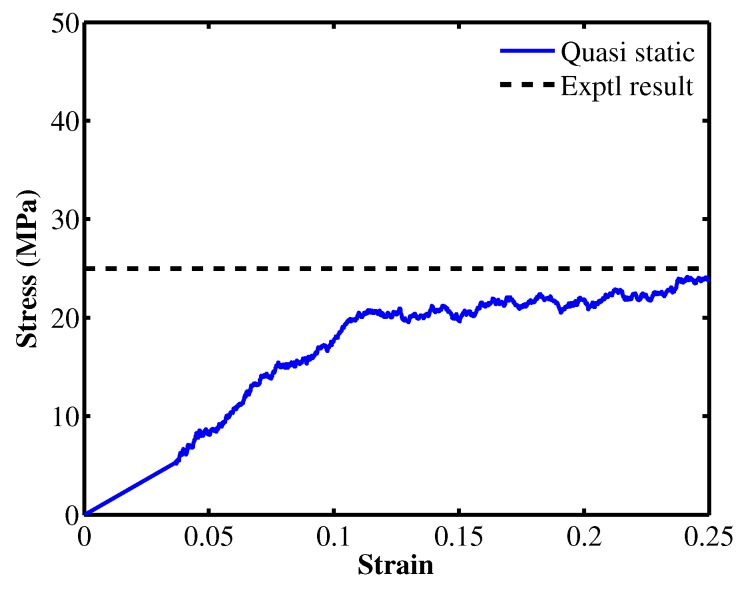
System of PE consisting of 40 PE molecules.

From the computational point of view, the minimization problem is equivalent to the equilibration problem. Therefore, we adopted a technique used to model quasi-static simulations proposed by Capaldi et al. [13] in this study. At first a constant strain rate of $10^{-5}$ 1/fs is applied to stretch the simulation box uniaxially for 1000 steps. The system is equilibrated for 10,000 steps (Δt=1 fs) subsequently. During the equilibration process, the box’s size in the stretched direction is kept fixed while the other box’s surfaces are subjected to NPT ensemble. The deformation-equilibration process is iterated until the desired deformation is achieved. The stress-strain response under quasi-static deformation is also plotted in Figure 5. The numerical result shows a good agreement with the experimental value reported in [11].

**Figure 6:**
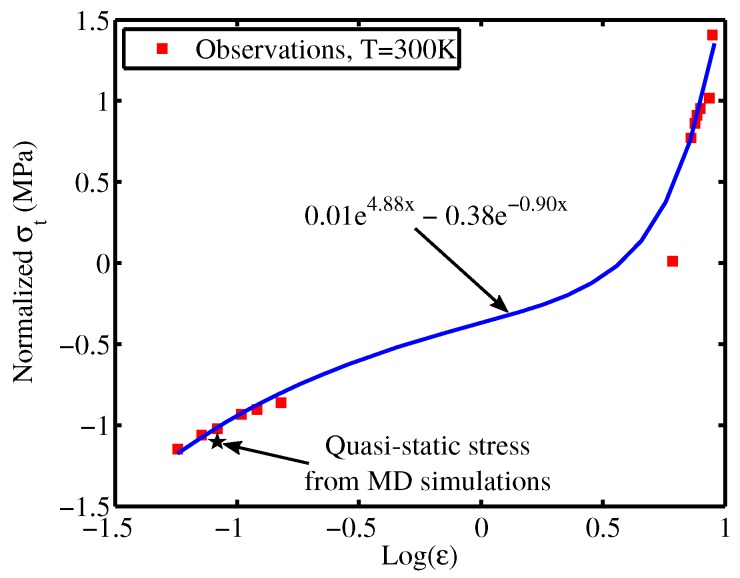
The extended constitutive law for the PE for the strain rate ranging from low (experiments) to high (MD simulations) values. The red solid squares represent the obtained tensile yield stresses from MD simulations (the group of values on the right) and experiments (the group of values on the left). The blue solid line is the fitted curve constructed based on the Bayesian method, see Section 4.2 in the manuscript. The black solid star represents the tensile yield stress under quasi-static deformation.

Since MD simulations are implemented at high strain rates while experiments are performed at low strain rates, a proper scaling law for the yield stress have to be constructed [2]. From the quasi-static MD simulations, the tensile yield stress at quasi-static strain rates is obtained. Then, Bayesian method is employed to construct the scaling law, as shown in Figure 6, extending from low (experiments) to high (MD simulations) strain rates for the PE.

For stochastic modeling, the temperature and strain rate are considered as random field. The temperature value is chosen lower than the glass transition temperature $T_{g}$ to ensure the glassy state of the PE while it should be high enough to ensure the strain rate affecting the mechanical properties can be observed [13]. Brown and Clarke [14] indicated that above 100 K, the Young’s modulus decreases significantly with an increase in temperature. In this study, we choose the temperature range from 100 to 300 K, though the $T_{g}$ is 280 K obtained from the cooling simulations, see Figure 2. Furthermore, we assume uniform distribution to characterize for the temperature parameter. The strain rate value ranged from $5\times 10^{-7}$ to $10^{-5}$ 1/fs are selected to perform stochastic modeling. We assume a uniform distribution to describe this parameter as a random field. The stress-strain curves in Figure 4a show that the Young’s modulus and the yield stress increase with an increase in strain rate at a fixed temperature while increasing the temperature results in a decrease in the Young’s modulus and the yield stress as illustrated in Figure 4b.

## 3. Stochastic Modeling

Uncertainty analysis aims to quantitatively assess the degree of confidence of models in which assumptions are usually made to predict the mechanical properties of materials based on available information. For models with multiple input parameters, using Monte-Carlo sampling (MCS) to generate random values is not efficient as the sample points are distributed uniformly. Instead Latin Hypercube Sampling (LHS) which is an efficient method [15] proposed by Iman et al. [16] can be used to generate samples for multivariate models based on known (or assumed) probability density function (PDF) of model inputs. To this end, the cumulative distribution function (CDF) of each input parameters of a multivariate *k* input models is split into *N* subdivisions equally, a N×k design matrix is then constructed where randomly independent values are dispersed uniformly. Subsequently, the input samples are inserted into the mechanical model to obtain the model outputs. By doing so, the mean and variance of the model outputs can be assessed, see [17] for details.

## 4. Surrogate Models

Sensitivity analysis requires a large number of samples to ensure reliable indices. It is impractical to measure the sensitivity indices based on the mechanical model. Alternative method can be used to reduce the computational expense is surrogate models as presented in [18]. The surrogate models using Kriging regression, and Bayesian updating methods will be employed in this study for performing sensitivity analysis.

### 4.1. Kriging Regression

#### 4.1.1. Maximum Likelihood Estimation

In this section, a general technique named Maximum likelihood estimation (MLE) is presented. It is used to estimate the statistical parameters of a mathematical model. Let consider a model with y being the model output vector characterized by a probability density function (PDF) $P_{y}\left(y;\theta \right)$ where θ denotes a vector of parameters that need to be estimated. The likelihood and its logarithmic function can be written as follow [19]:

(1) $$L=\frac{1}{{\left(2\;\pi \;\sigma^{2}\right)}^{N/2}\;\sqrt{\det(\Psi )}}\;\exp\left[-\frac{{\left(y-1\;\mu \right)}^{T}\;\Psi^{-1}\;\left(y-1\;\mu \right)}{2\;\sigma^{2}}\right],$$

and

(2) $$\ln L\approx -\frac{N}{2}\;\ln\left(\sigma^{2}\right)-\frac{1}{2}\;\ln\left|\Psi \right|,$$

where the MLEs of μ and $\sigma^{2}$ are given by

(3) $$\hat{\mu }=\frac{1^{T}\;{\left(\Psi +\lambda \;I\right)}^{-1}\;y}{1^{T}\;{\left(\Psi +\lambda \;I\right)}^{-1}\;1},\;{\hat{\sigma }}^{2}=\frac{{\left(y-1\;\hat{\mu }\right)}^{T}\;{\left(\Psi +\lambda \;I\right)}^{-1}\;\left(y-1\;\hat{\mu }\right)}{N},$$

with λ being the positive regression constant and lnL becomes

(4) $$\ln L\approx -\frac{N}{2}\;\ln\left(\sigma^{2}\right)-\frac{1}{2}\;\ln\left|\Psi +\lambda \;I\right|.$$

It is worth noting that the parameters $\theta_{i}$ and λ are estimated by maximizing lnL.

#### 4.1.2. Kriging Prediction

Considering a multivariate model whose input vector is $x={\left\{x_{1},\;x_{2},\;...,\;x_{N}\right\}}^{T}$ and measurement vector is $y={\left\{y_{1},\;y_{2},\;...,\;y_{N}\right\}}^{T}$ with *N* being the number of training data. If we express the measurement vector as a function of the input vector $y={\left\{y\left(x_{1}\right),\;y\left(x_{2}\right),\;...,\;y\left(x_{N}\right)\right\}}^{T}$, a regression model that approximates the training data can be found to predict $\hat{y}$ at an arbitrary point $x_{m}$ within the input ranges. Assuming the variables to correlate to each other through the anisotropic exponential functions shown by:

(5) $$\mathrm{cor}\left(Y\left(x_{𝚤}\right),Y\left(x_{𝚥}\right)\right)=\sigma^{2}\exp\left(-\sum_{i=1}^{k}\theta_{i}{\left(x_{𝚤i}-x_{𝚥i}\right)}^{2}\right),\;𝚤,\;𝚥=1,...,N.$$

with $\sigma^{2}$ being the variance of the measurement at training points, and $\theta_{i}$ being a parameter characterizing the degree of correlation in $i^{th}$ direction between one parameter with another one. The covariance matrix of all training points can be shown by:

(6) $$\Psi =\left(\begin{array}{ccc} \mathrm{cor}(y\left(x_{1}\right),y\left(x_{1}\right)) & \cdots & \mathrm{cor}(y\left(x_{1}\right),y\left(x_{N}\right))\\ \vdots & \ddots & \vdots \\ \mathrm{cor}(y\left(x_{N}\right),y\left(x_{1}\right)) & \cdots & \mathrm{cor}(y\left(x_{N}\right),y\left(x_{N}\right)) \end{array}\right).$$

Maximizing the logarithmic likelihood lnL in Equation (4), we can obtain the parameters $\mu ,\;\sigma^{2}$, θ,andλ. A gradient free optimization technique, e.g., a genetic algorithm (GA) method, can be used to evaluate these parameters, see [20]. If a correlation vector between the measurement and the new prediction is defined as

(7) $$\psi =\left(\begin{array}{c} \mathrm{cor}(y\left(x_{1}\right),y\left(x\right))\\ \vdots \\ \mathrm{cor}(y\left(x_{N}\right),y\left(x\right)) \end{array}\right)=\left(\begin{array}{c} \psi_{1}\\ \vdots \\ \psi_{N} \end{array}\right).$$

The prediction at the new point $x_{m}$ can be subsequently estimated by

(8) $$\hat{y}\left(x\right)=\hat{\mu }+\psi^{T}{\left(\Psi^{-1}+\lambda I\right)}^{-1}\left(y-1\hat{\mu }\right).$$

### 4.2. Bayesian Updating

Alternative method—Bayesian approach—can be used to identify the parameters for the stochastic constitutive model (surrogate model) [1]. In this method, we assume a *prior distribution p(θ)* for the random parameter θ at first. Then, it will be estimated considering the model evidence as follows:

(9) $$p\left(\theta |z\right)=\frac{p(z|\theta )p(\theta )}{p(z)},$$

where θ,z denote the vector of model parameters and the vector of measurements. The term p(z) can be skipped for purpose of parameter identification while the posterior p(θ|z) can be written in terms of a proportional product of likelihood p(z|θ) and the prior p(θ) shown by:

(10) $$\underset{\mathrm{posterior}}{\underset{︸}{p(\theta |z)}}\propto \underset{\mathrm{likelihood}}{\underset{︸}{p(z|\theta )}}\underset{\mathrm{prior}}{\underset{︸}{p(\theta )}}.$$

Given data the maximum a posterior (MAP) probability of the parameters is then determined by:

(11) $$\theta_{\mathrm{MAP}}=\underset{\theta }{\mathrm{argmax}}\;p\left(z|\theta \right)p\left(\theta_{i}\right)$$

## 5. Sensitivity Analysis

In this section, a global SA method—variance based method—presented by Saltelli et al. [21] is used to measure how much the model output varies when varying the input parameters. Based on this method, contribution of the input parameters to the model output will be quantitatively assessed.

### 5.1. First-Order Sensitivity Indices

Recalling the multivariate *k*-input model, the measurement vector can be expressed by $y=f(x_{1},\;x_{2},...,\;x_{k})$. The first order index are given by [22,23]

(12) $$S_{i}=\frac{V_{x_{i}}\left[E_{x_{∼}i}\left(y|x_{i}\right)\right]}{V(y)},$$

in which V(y) denote the unconditional variance of y, $E_{x_{∼}i}\left(y|x_{i}\right)$ denote the variance of the mean value E(y) when keeping $x_{i}$ fixed and $V_{x_{i}}\left[E_{x_{∼}i}\left(y|x_{i}\right)\right]$ is its variance which estimates the main effect of the parameter $x_{i}$ on the model output.

### 5.2. Total Effect Sensitivity Indices

Since a part of variance of the output resulting from the variance of the input parameter $x_{i}$ is evaluated by the first order index, higher order indices of coupling terms need to be extended to evaluate the total variance of the output. Therefore, the total effect $S_{Ti}$ is used to assess the contribution of the input parameter $x_{i}$ to the output’s variance. The total effect index is given as follows [24]

(13) $$S_{Ti}=1-\frac{V_{x_{∼i}}\left[E_{x_{i}}\left(y|x_{∼i}\right)\right]}{V(y)},$$

in which $E_{x_{i}}\left(y|x_{∼i}\right)$ is the mean value of y when keeping all parameters but $x_{i}$ fixed and $V_{x_{∼i}}\left[E_{X_{i}}\left(y|x_{∼i}\right)\right]$ is its variance. Note that the later shows the main effect of $x_{∼i}$ on the output. Also, the difference between $S_{Ti}$ and $S_{i}$ indicates the level of interaction of $x_{i}$ with other input parameters.

**Figure 7:**
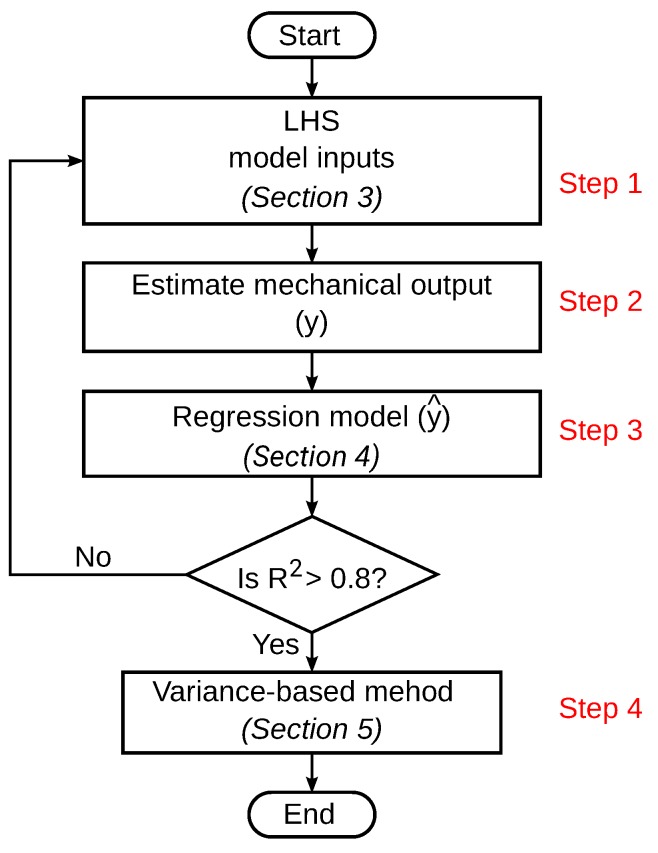
Flow chart for numerical estimation of the effect of the input parameters on the model output.

Numerical procedure for estimation of the effect of the input parameters on the model outputs is summarized by the flowchart shown in Figure 7.

## 6. Numerical Results

In the numerical examined here, we consider the effect of the two input parameters, i.e., temperature ($X_{2}$) and strain rate ($X_{3}$) on either the yield stress or the elastic modulus obtained from simulations presented in Section 2. Since the computational expense of the fully atomistic model are highly expensive while the computation of the first order and the total effect requests a large number of samples (model runs), surrogate models will be employed to replace the mechanical model. The quadratic polynomial function $y=\beta_{1}x_{1}+\beta_{2}x_{2}+\beta_{3}x_{1}^{2}+\beta_{4}x_{2}^{2}$ is used in the scope of Bayesian updating approach. Subsequently, the SA will be performed based on the surrogate models. It should be noted that surrogate model serves as a vehicle in which uncertainty is taken to a set of phenomenological constitutive law parameters (i.e. the temperature and the strain rate).

**Figure 8:**
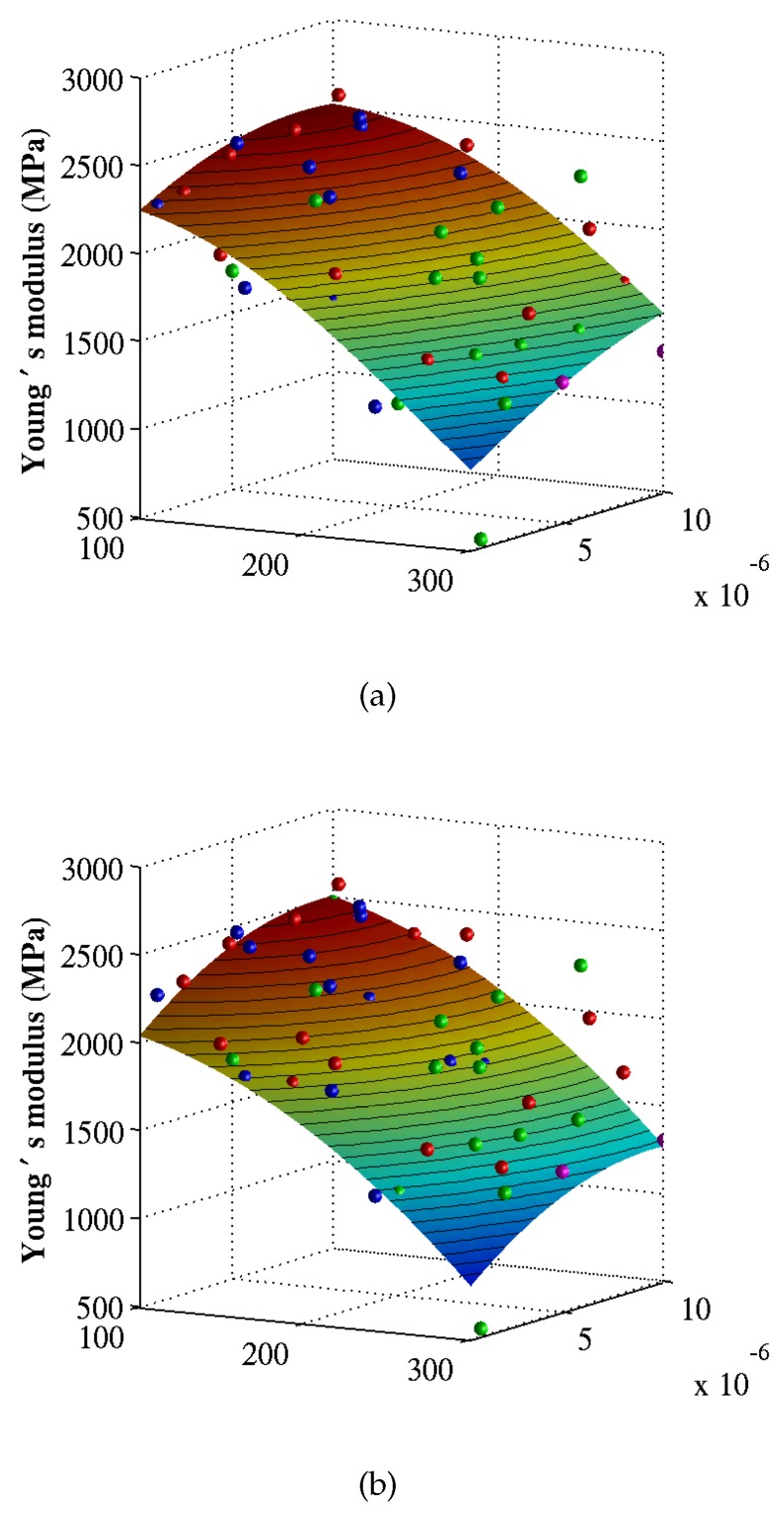
Surrogate models for the Young’s modulus corresponding to (**a**) Kriging regression and (**b**) Bayesian updating. The training data are obtained from MD simulations.

**Figure 9:**
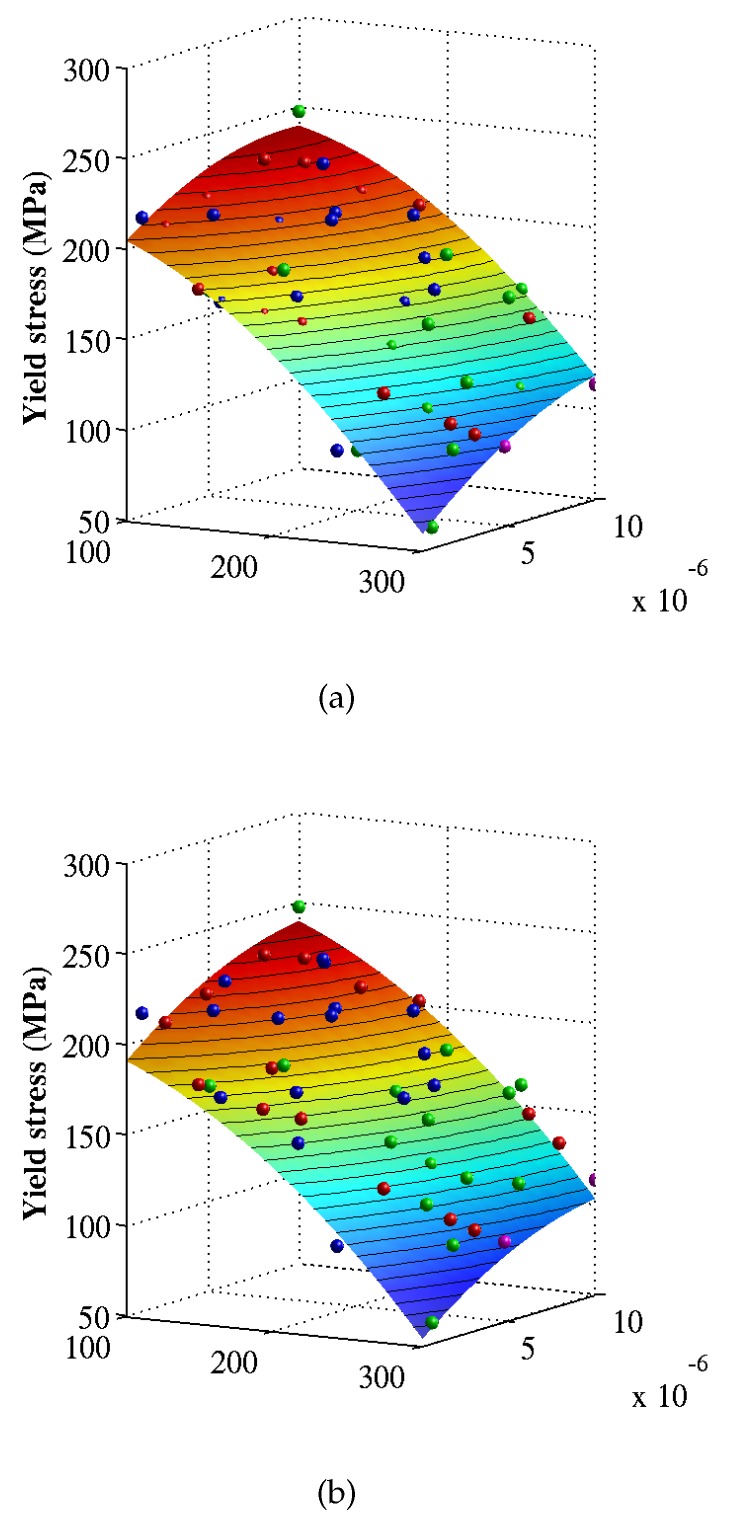
Surrogate models for the yield stress corresponding to (**a**) Kriging regression and (**b**) Bayesian updating. The training data are obtained from MD simulations.

Figure 8 and Figure 9 shows the surrogate models constructed using the Kriging regression and Bayesian updating methods for the Young’s modulus and the yield stress, respectively. The highest gradient occur in temperature direction, showing the temperature as an important parameter for the yield stress. The parameters and the coefficient of determination (COD) of the surrogates models are illustrated in Table 2 and Table 3 in accordance with the Kriging and the Bayesian updating approaches, respectively. Based on the COD, we realize that Kriging regression provides better approximation than Bayesian updating does for the Young’s modulus. Therefore, the Kriging regression method will be used to construct the surrogate model on which the sensitivity indices are measured.

**Figure 10:**
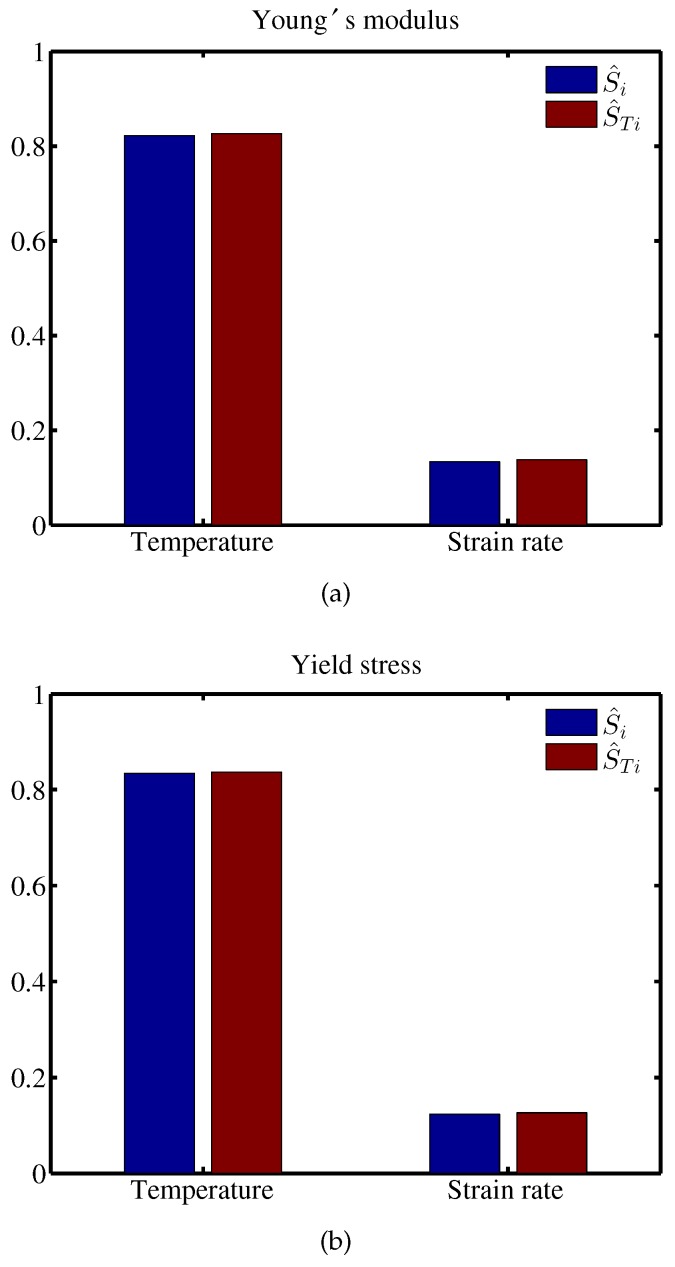
First order and total effect indices for (**a**) the Young’s modulus and (**b**) the yield stress using Kriging regression.

We generated $10^{4}$ samples using LHS approach. The respective sensitivity indices $S_{i}$ and $S_{Ti}$, shown in Equations (12) and (13), are then computed based on the Kriging regression model and illustrated in Figure 10. The sensitivity indices presented in Table 4 and Table 5 show that the temperature is the key parameter that affects the Young’s modulus and the yield stress the most. Furthermore, the equivalence between the first order and the total effect indices indicates that the temperature and the strain rate parameters are independent of each other. Note that these indices are reduced by the COD that infers that only $R^{2}\%$ of the mechanical properties is approximated by the surrogate model.

## 7. Conclusions

In this study, MD simulations are used to construct the stochastic constitutive models using Kriging regression and Bayesian updating approaches. Uncertainty quantification is also performed based on the surrogate models (i.e., the stochastic constitutive models) via SA. The influence of the temperature and the strain rate is then quantified. From the sensitivity indices, the temperature shows a pronounced influence on the Young’s modulus and the yield stress compared to the strain rate.

## Figures and Tables

**Table 1:** Mechanical properties of the PE (units in GPa, K and $g/{\mathrm{cm}}^{3}$).

Tension		
Constant	MD	Experimental results
	simulations	HPDE
Young’s modulus	1.22	1.18 [11]
Glass transition temperature	280	250 [9], 255 [10] and 280 [13]

**Table 2:** Kriging regression.

Property	$\theta_{1}$	$\theta_{2}$	λ	$R^{2}$
Young’s modulus	0.03	0.02	0.03	0.96
Yield stress	0.03	0.01	0.005	0.96

**Table 3:** Bayesian updating for model $y=\beta_{1}x_{1}+\beta_{2}x_{2}+\beta_{3}x_{1}^{2}+\beta_{4}x_{2}^{2}$.

Property	$\beta_{1}$	$\beta_{2}$	$\beta_{3}$	$\beta_{4}$	$R^{2}$
Young’s modulus	−0.85	0.35	−0.15	−0.12	0.82
Yield stress	−0.94	0.35	−0.14	−0.08	0.96

**Table 4:** First order and total effect sensitivity indices for the Young’s modulus using Kriging regression.

Index	$X_{1}$	$X_{2}$	$X_{3}$	$X_{4}$
${\hat{S}}_{i}$	0.92	0.76	0.29	0.17
${\hat{S}}_{Ti}$	0.13	0.02	0.00	0.00

**Table 5:** First order and total effect sensitivity indices for the Young’s modulus using Bayesian updating.

Index	$X_{1}$	$X_{2}$	$X_{3}$	$X_{4}$
${\hat{S}}_{i}$	0.92	0.76	0.29	0.17
${\hat{S}}_{Ti}$	0.13	0.02	0.00	0.00
